# Whole-Brain Wiring Diagram of Oxytocin System in Adult Mice

**DOI:** 10.1523/JNEUROSCI.0307-22.2022

**Published:** 2022-06-22

**Authors:** Seoyoung Son, Steffy B. Manjila, Kyra T. Newmaster, Yuan-ting Wu, Daniel J. Vanselow, Matt Ciarletta, Todd E. Anthony, Keith C. Cheng, Yongsoo Kim

**Affiliations:** ^1^Department of Neural and Behavioral Sciences, The Pennsylvania State University, Hershey, Pennsylvania 17033; ^2^Department of Pathology, The Pennsylvania State University, Hershey, Pennsylvania 17033; ^3^Program in Neuroscience, Harvard University, Boston, Massachusetts 02138

**Keywords:** anatomical connectivity, axonal output, brain mapping, oxytocin, synaptic input, wiring diagram

## Abstract

Oxytocin (Oxt) neurons regulate diverse physiological responses via direct connections with different neural circuits. However, the lack of comprehensive input-output wiring diagrams of Oxt neurons and their quantitative relationship with Oxt receptor (Oxtr) expression presents challenges to understanding circuit-specific Oxt functions. Here, we establish a whole-brain distribution and anatomic connectivity map of Oxt neurons, and their relationship with Oxtr expression using high-resolution 3D mapping methods in adult male and female mice. We use a flatmap to describe Oxt neuronal expression in four hypothalamic domains including under-characterized Oxt neurons in the tuberal nucleus (TU). Oxt neurons in the paraventricular hypothalamus (PVH) broadly project to nine functional circuits that control cognition, brain state, and somatic visceral response. In contrast, Oxt neurons in the supraoptic (SO) and accessory (AN) nuclei have limited central projection to a small subset of the nine circuits. Surprisingly, quantitative comparison between Oxt output and Oxtr expression showed no significant correlation across the whole brain, suggesting abundant indirect Oxt signaling in Oxtr-expressing areas. Unlike output, Oxt neurons in both the PVH and SO receive similar monosynaptic inputs from a subset of the nine circuits mainly in the thalamic, hypothalamic, and cerebral nuclei areas. Our results suggest that PVH-Oxt neurons serve as a central modulator to integrate external and internal information via largely reciprocal connection with the nine circuits while the SO-Oxt neurons act mainly as unidirectional Oxt hormonal output. In summary, our Oxt wiring diagram provides anatomic insights about distinct behavioral functions of Oxt signaling in the brain.

**SIGNIFICANCE STATEMENT** Oxytocin (Oxt) neurons regulate diverse physiological functions from prosocial behavior to pain sensation via central projection in the brain. Thus, understanding detailed anatomic connectivity of Oxt neurons can provide insight on circuit-specific roles of Oxt signaling in regulating different physiological functions. Here, we use high-resolution mapping methods to describe the 3D distribution, monosynaptic input and long-range output of Oxt neurons, and their relationship with Oxt receptor (Oxtr) expression across the entire mouse brain. We found Oxt connections with nine functional circuits controlling cognition, brain state, and somatic visceral response. Furthermore, we identified a quantitatively unmatched Oxt-Oxtr relationship, suggesting broad indirect Oxt signaling. Together, our comprehensive Oxt wiring diagram advances our understanding of circuit-specific roles of Oxt neurons.

## Introduction

Oxytocin (Oxt) is a highly conserved neuropeptide, playing key roles in regulating social behavior and other physiological functions (Althammer et al., 2018; Jurek and Neumann, 2018; Quintana and Guastella, 2020). Impairment in Oxt signaling has been heavily implicated in many neurodevelopmental disorders including autism (Francis et al., 2014; Rajamani et al., 2018). Altering Oxt signaling is being pursued as a potential therapy to alleviate social behavioral deficits in many brain disorders (Guastella and Hickie, 2016). However, our limited neural circuit based understanding of Oxt signaling in the brain hampers the development of targeted therapeutic approaches aimed at altering specific Oxt functions without affecting other biological pathways. A comprehensive anatomic understanding of Oxt neurons would enable integrated neural circuit-specific studies to decipher the neural substrate of distinct Oxt functions.

The majority of Oxt producing neurons are located in the paraventricular nucleus of the hypothalamus (PVH) and the supraoptic nucleus (SO) while fewer Oxt neurons reside in the extended amygdala (Biag et al., 2012; Madrigal and Jurado, 2021). Oxt neurons receive input from distinct brain regions (e.g., the thalamus) and integrate sensory input with internal information to release Oxt in a context dependent manner to modulate specific downstream circuitry (Tang et al., 2020; Grinevich and Neumann, 2021). The actions of Oxt are mainly mediated by a single subtype of the Oxt receptor (Oxtr) expressed in distinct brain regions as well as peripheral tissues (Gimpl and Fahrenholz, 2001; Grinevich et al., 2016; Newmaster et al., 2020). In addition to the well-known peripheral release of Oxt as a hormone via the posterior pituitary, Oxt neurons send direct projections to specific brain areas that frequently express Oxtr, thereby modulating circuit-specific functions (Grinevich et al., 2016; Liao et al., 2020). For example, Oxt signaling is linked with the medial prefrontal cortex for social cognition (Sabihi et al., 2014; Li et al., 2016), CA2 of the hippocampus for social memory (Raam et al., 2017; Tirko et al., 2018), the central amygdala for fear modulations (Knobloch et al., 2012; Ferretti et al., 2019), the parabrachial nucleus (PB) for fluid intake (Ryan et al., 2017), and the spinal cord for pain perception (Eliava et al., 2016; Boll et al., 2018). Despite these prior studies, we still lack a quantitative and comprehensive wiring diagram of the Oxt neurons in a standard 3D reference brain.

Here, we establish a comprehensive wiring diagram of Oxt neurons in the mouse brain using a high-resolution quantitative brain mapping method in combination with cell type-specific transgenic mice and viral tools. All whole-brain datasets are registered in the Allen Common Coordinate Framework (CCF) to facilitate data cross-comparison (Wang et al., 2020), and high-resolution images can be easily viewed using a new web visualization (https://kimlab.io/brain-map/ot_wiring/). Using the new resource, we identified distinct Oxt neuronal connection with nine circuits that can explain diverse Oxt functions. Moreover, we found lack of quantitative correlation between Oxt output and Oxtr expression across the whole brain, suggesting abundant indirect Oxt signaling in Oxtr-expressing brain areas.

## Materials and Methods

### Animals

All animal care and experimental procedures are approved by the Penn State University Institutional Animal Care Use Committee (IACUC). Oxt-Cre mice (Choe et al., 2015) were originally produced in the Gloria B. Choi lab at the Massachusetts Institute of Technology and imported to the Penn State University (Kim Lab). To generate Oxt-Cre;Ai14 mice, Oxt-Cre mice were crossed with Ai14 mice, expressing tdTomato following Cre-mediated recombination (Jax: 007914, C57Bl/6 J background). Oxtr-Venus mice were imported from the Nishimori Lab in Tohoku University that created the line (Yoshida et al., 2009). Two-month-old C57Bl/6 J mice were used for whole-brain tissue clearing and immunostaining. Mice received food and water *ad libitum* and were housed under constant temperature and light conditions (12/12 h light/dark cycle).

### Experimental design and statistical analyses

**Figure 1.**
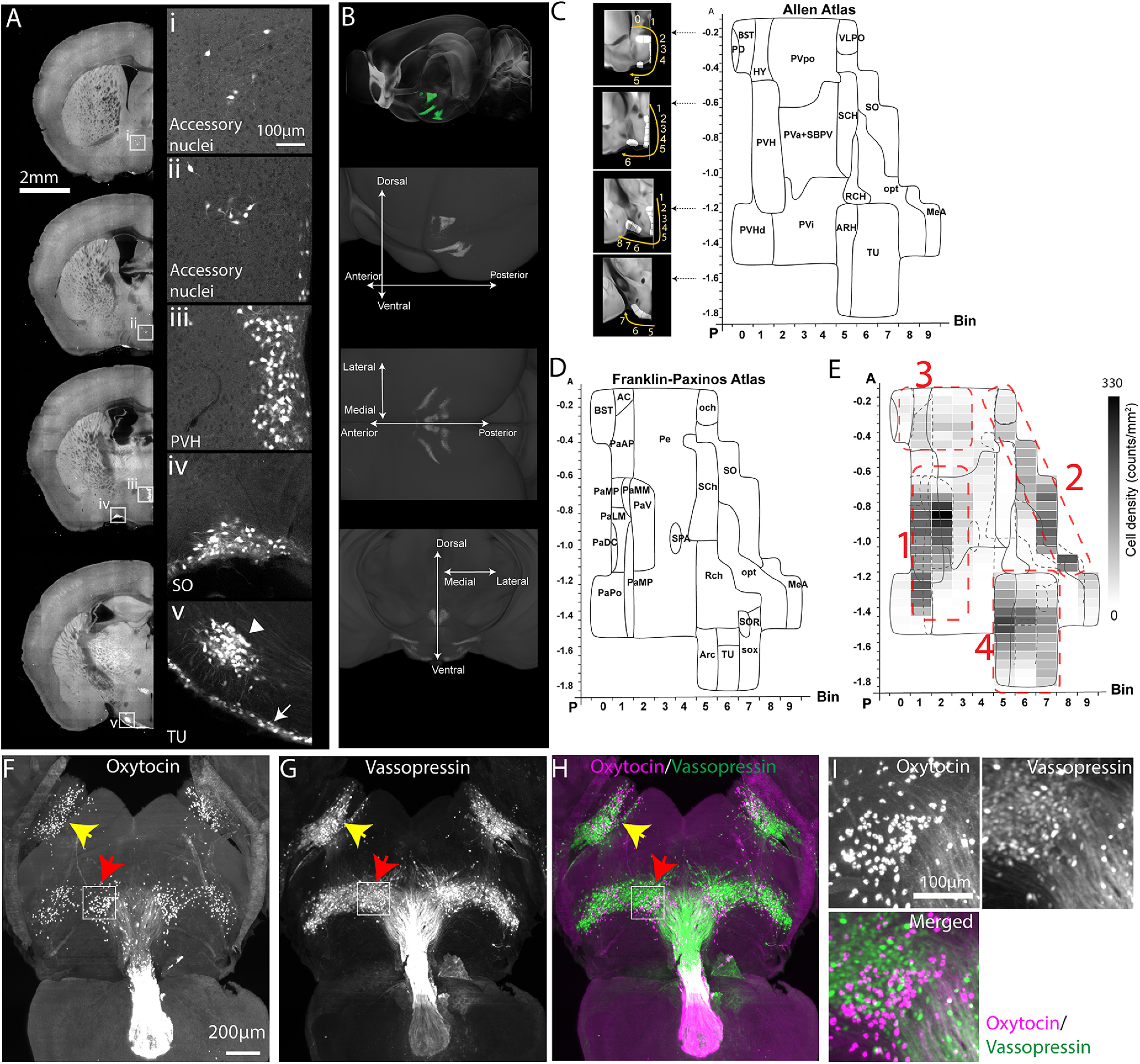
Distribution of Oxt neurons. ***A***, Signals from Oxt-Cre;Ai14 mice across representative coronal planes of the mouse brain. Figures on the right column are high-magnification images from white boxed areas in the left column. The TU (in v panel) contains two subpopulations of Oxt neurons with a small cluster (arrowhead) and a thin layer along the bottom edge of the brain (arrow). Please see Extended Data Figure 1-1 for Oxt antibody validation of Oxt-Cre;Ai14 mice. ***B***, Top, STPT images registered onto the Allen CCF reference brain. Green signals represent averaged Oxt neurons from 8 animals. (bottom) 3D distribution of Oxt neurons. See also Movie 1, Table 1, and Extended Data Table 1-1. ***C***, ***D***, 2D hypothalamic flatmaps. Small insets with coronal sections illustrate how bins (while areas with numbers) were generated at different coronal planes. Anatomical labels in the flatmap are delineated based on Allen mouse brain atlas (***C***) and Franklin–Paxinos atlas (***D***). The *x*-axis is for bin numbers and *y*-axis is for the bregma A/P axis. The full name of abbreviations can be found in Table 1. ***E***, Heatmap of Oxt neuronal density in four clusters with the overlay of Allen and Franklin–Paxinos labels in solid and dotted lines, respectively. Red dotted lines for four Oxt-expressing domains. 1: PVH, 2: SO, 3: AN, and 4: TU. ***F–I***, LSFM imaging of whole-brain immunostaining with Oxt and vasopressin antibodies; 500-µm-thick z maximum projection of Oxt (***F***), vasopressin (***G***), and both (***H***). Yellow and red arrows for the SO and the TU, respectively. ***I***, High-magnification images from the white boxed TU area in ***F***, ***G***. Note the lack of colocalization between the Oxt and vasopressin.

**Figure 2.**
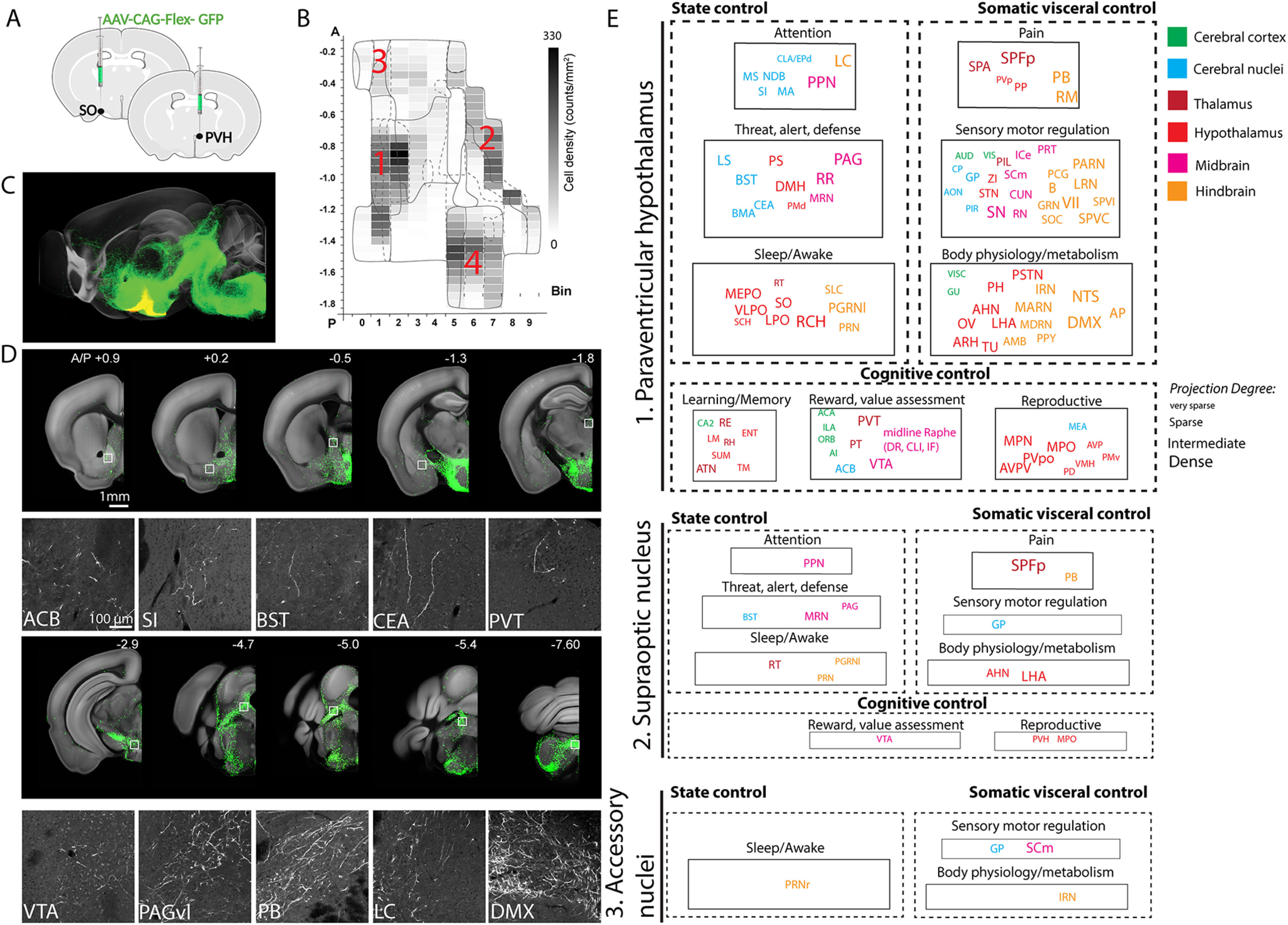
Anterograde projection map of Oxt neurons. ***A***, Conditional AAV-GFP was injected in Oxt neuron containing hypothalamic areas. ***B***, Four major areas of viral injections, 1: the PVH, 2: the SO, 3: the AN, 4: the TU area. ***C***, Projection outputs from the PVH (green) and SO (yellow) Oxt neurons registered in the Allen CCF. See also Movie 2. ***D***, Examples of long-range projections (green) from Oxt neurons in the PVH. The bottom panel is high mag images from white boxed areas in the top panel. ***E***, Nine functional circuits that receive long-range projection from Oxt neurons in the three different injection area 1–3. Color and size of each ROI represent anatomic ontology and the abundance (degree) of the projection. The full name of abbreviations can be found in Table 2.

**Figure 3.**
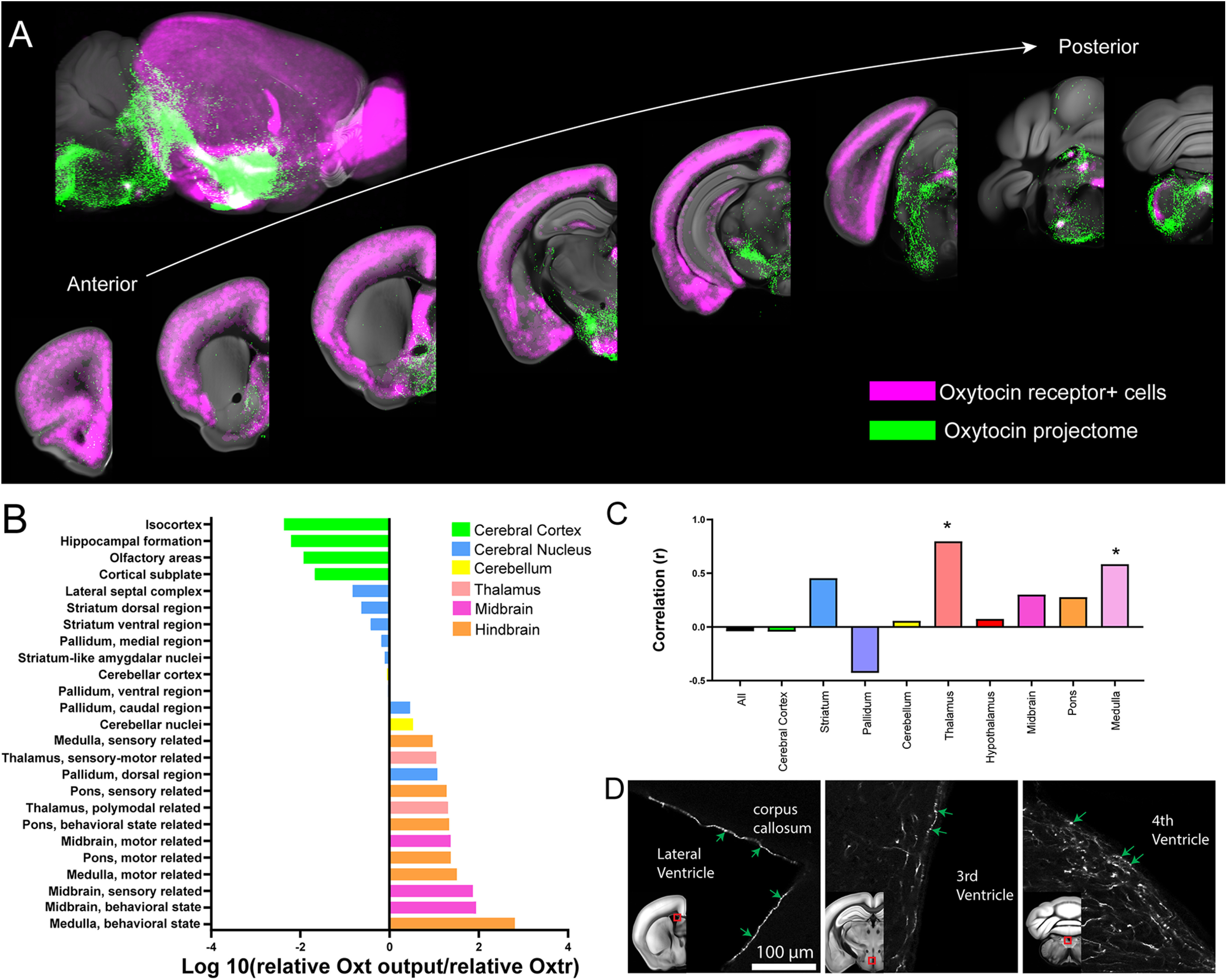
Comparison between the Oxt output and Oxtr expression. ***A***, Composite images of representative Oxt neuronal projection outputs (red: combined from both the PVH and the SO) and Oxtr expression (green) across the mouse brain. See also Movie 3. ***B***, Quantitative comparison of relative Oxt projection pattern and Oxtr expression. Note that the cerebral cortex has very small Oxt/Oxtr ratio while the hindbrain and the midbrain shows higher ratio. ***C***, Correlation between Oxt projection and Oxtr density (Spearman nonparametric correlation, *p < 0.05). Note no significant correlation across the whole-brain areas despite the significant correlation in the thalamus and the medulla. ***D***, Examples of Oxt long range projection touching the surface of all major ventricles.

**Figure 4.**
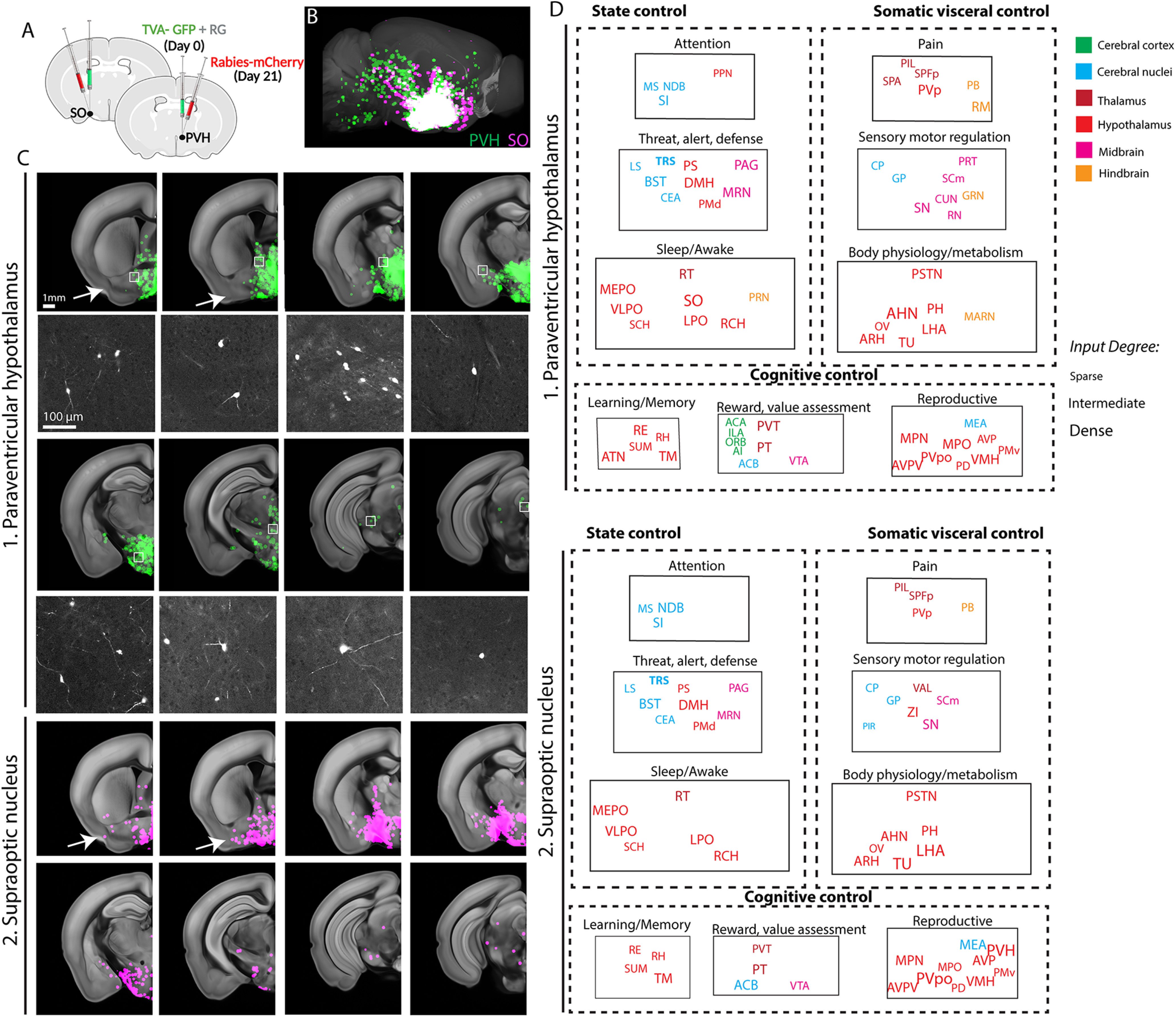
Monosynaptic input map of Oxt neurons in the PVH and the SO. ***A***, Conditional monosynaptic tracing rabies virus was injected in the PVH or the SO of the Oxt-Cre mice. See Extended Data Figures 4-1 and 4-2 for control experiments to support cell type specificity of the rabies tracing. ***B***, Brain-wide inputs into the PVH (green, *n* = 6 animals) and SO (red, *n* = 4 animals) Oxt neurons. The maximum signals of all samples from each anatomic region were overlaid on the reference brain. See also Movie 4. ***C***, Representative monosynaptic inputs in different coronal planes (top panel) and high mag images from white boxed areas (bottom panel). Arrows highlight input from lateral brain areas for the SO compared with the PVH. ***D***, Nine functional circuits that provide monosynaptic input to Oxt neurons in the two anatomic areas. Note overall similar input patterns for both areas. The full name of abbreviations can be found in Table 2.

For Oxt neuron distribution mapping (Fig. 1, Movie 1), we used three males, three females (virgin), and two females (lactating) of two- to four-month-old Oxt-Cre;Ai14 mice with serial two-photon tomography (STPT) imaging. We also used four males, three females (virgin) of two-month-old C57bl/6 mice for tissue clearing and light sheet fluorescence microscopy (LSFM) imaging-based quantification (Fig. 1). Since we did not observe significant difference in Oxt neuronal number, we combined data from both sexes to generate representative cell counting (Table 1; Extended Data Table 1-1). For anterograde projectome mapping in two- to four-month-old Oxt-Cre (Fig. 2), we used two males, three females (virgin), and three females (lactating) with 500 nl of AAV injection, and three males, three females (virgin), and one female (lactating) with 50–150 nl of AAV injection for the PVH targeting. Moreover, we used five males and five females (virgin) for the SO, three males and two females (virgin) for the TU, one male and one female (virgin) for the AN. For Oxtr expression mapping using Oxtr-Venus mice (Fig. 3), we used six males and eight females (virgin). For rabies input mapping (Fig. 4), we used three males and three females (virgin) for the PVH, and three males and one female (virgin) for the SO.

**Table 1.** Oxt neuron counting

Cluster	Full names	Abbreviations	Transgenic	3D immunolabeling
1	Paraventricular hypothalamic nucleus	PVH	511.5 ± 147.1	818 ± 169
	Periventricular hypothalamic nucleus, anterior part	PVa	47.6 ± 22.7	119 ± 23
	Periventricular hypothalamic nucleus, intermediate part	PVi	9.6 ± 11.4	14 ± 5
	Subparaventricular zone	SBPV	17.9 ± 20.8	113 ± 49
	Paraventricular hypothalamic nucleus, descending division	PVHd	153.8 ± 49.7	27 ± 16
2	Supraoptic nucleus	SO	202.3 ± 65.5	654 ± 89
	Medial amygdalar nucleus	MEA	108.7 ± 49.6	10 ± 4
	Ventrolateral preoptic nucleus	VLPO	21.9 ± 23.3	50 ± 20
3	Bed nuclei of the stria terminalis	BST	27.8 ± 11	68 ± 34
	Periventricular hypothalamic nucleus, preoptic part	PVpo	51.1 ± 18.3	194 ± 40
	Substantia innominata	SI	6 ± 3.1	2 ± 2
	Medial preoptic nucleus	MPN	18 ± 5.8	71 ± 14
	Lateral hypothalamic area	LHA	45.9 ± 14.4	32 ± 12
	Lateral preoptic area	LPO	2.1 ± 2	4 ± 2
4	Tuberal nucleus	TU	472.9 ± 65.2	393 ± 121
	Arcuate hypothalamic nucleus	ARH	147.5 ± 86.5	272 ± 83

Transgenic animal counting is from STPT imaging of Oxt-Cre;Ai14 mice (*n* = 8) and 3D immunolabeling counting is from LSFM imaging of C57 after tissue clearing and 3D Oxt antibody staining (*n* = 7). Counting data are mean ± SD. Cell counting data from male, virgin female, and lactating females can be found in Extended Data Table 1-1.

10.1523/JNEUROSCI.0307-22.2022.tab1-1 Extended Data Table 1-1 **Comparison of Oxt cell counts across males, virgin females, and lactating females** Download Table 1-1, DOCX file.

To determine the correlation between Oxt area normalized projection and Oxtr density (Fig. 3*C*), we first tested for the normality of the data using the D'Agostino–Pearson normality test. Based on the normality test result, we performed Spearman nonparametric correlation test. GraphPad Prism 8 was used for all statistical analysis and graphs.

### Stereotaxic surgery and virus injections

Oxt-Cre mice (8–11 weeks old, males and females) were anesthetized with isoflurane (controlled with Somnosuite, Kent Scientific) and mounted on a stereotaxic instrument (Angle Two, Leica) with a heating pad placed underneath. All injections were performed with pulled micropipettes (VWR, catalog #53432-706). Through the small opening of the micropipette, virus was delivered at a rate of 75–100 nl/min. The speed and volume of injection were monitored along with the calibration marks on the micropipette (1 mm = 100 nl). To target the PVH, coordinates are anteroposterior (AP) from the bregma: −0.58 mm; mediolateral (ML): 0.27 mm; dorsoventral (DV): −4.75 mm. Anterior PVH and posterior PVH injection coordinates are −0.35 mm (AP), 0.3 mm (ML), and −4.5 mm (DV) and −0.94 mm (AP), 0.39 mm (ML), and −4.55 mm (DV), respectively. Coordinates for the SO are −0.66 mm (AP), 1.3 mm (ML), and −5.8 mm (DV). For anterograde tracing, 50–500 nl of AAV2-CAG-Flex-EGFP virus (titer 3.7 × 10^12^ vg/ml, purchased from UNC vector core) was injected into the PVH (500 nl for maximum coverage, 50–150 nl for PVH subregion) and 150 nl of the virus was injected into the SO. Mice were euthanized three weeks later with ketamine (100 mg/kg) and xylazine (10 mg/kg) mixture. For monosynaptic retrograde labeling, 50–500 nl of rAAV1-synp-DIO-STPEPB (titer 3.9 × 10^12^ vg/ml, purchased from UNC vector core, a gift from Ian Wickersham; Addgene plasmid #52473; http://n2t.net/addgene:52473; RRID:Addgene_52473; Kohara et al., 2014) was injected into the PVH, followed by the same quantity of EnvA G-deleted Rabies-mcherry virus [titer: 8.12 × 10^8^ TU/ml, purchased from the Salk Institute Viral Vector Core, a gift from Edward Callaway (Osakada et al., 2011), RRID:Addgene_32636] three weeks later into the same location. Mice were euthanized 7–8 d later with ketamine (100 mg/kg) and xylazine (10 mg/kg) mixture.

Control experiments were performed by injecting 500 nl of rAAV1/Synp-DIO-STPE (titer 4.3 × 10^12^ vg/ml, purchased from UNC vector core, a gift from Ian Wickersham (Addgene plasmid #52474; http://n2t.net/addgene:52474; RRID:Addgene_52474; Kohara et al., 2014) in Oxt-Cre mice at the same co-ordinates for PVH and SO (*N* = 1 each). EnvA G-deleted Rabies-mcherry virus was injected three weeks later and the mice were euthanized 7 d later for brain collection.

To check for leakiness of avian tumor receptor virus A (TVA), 500 nl of rAAV1-synp-DIO-STPEPB was injected along with 50 µl of 1:4 diluted pAAV-CAG-tdTomato (codon diversified; a gift from Edward Boyden (Addgene plasmid #59462; http://n2t.net/addgene:59462; RRID:Addgene_59462) in C57 mice at the same co-ordinates for PVH.

For the optimized G and split TVA tracing, AAV5-CAG(del)>TCIT(-ATG)-Flex(*loxP*)-SV40 (1:8 dilution, titer: 1.6E+12 gc/ml, a gift from Todd Anthony at Harvard) was co-injected with AAV5- CAG(del)>nC2oG-Flex(loxP) (titer: 2.8E+12 gc/ml, a gift from Todd Anthony) in Oxt-Cre mice (*N* = 2 each for PVH and SO co-ordinates; 150 nl per brain). After 14 d, the same volume of EnvA G-deleted Rabies-EGFP virus (titer: 4.89E+09 TU/ml, purchased from Salk Institute viral vector core, a gift from Edward Callaway; Addgene plasmid #32635; http://n2t.net/addgene:32635; RRID:Addgene_32635; Osakada et al., 2011) was injected into the same location. The mice were euthanized 7 d later for brain collection and STPT imaging.

### STPT imaging and related data analysis

Transgenic or virus injected mice were transcardially perfused with 4% paraformaldehyde (PFA) in 0.1 m phosphate buffer (PB; pH 7.4) after 0.9% saline. Brains were dissected out and postfixed in 4% PFA overnight at 4°C. Fixed brains were stored in 0.05 m PB at 4°C until imaged. To image the entire brain, STPT (TissueCyte 1000; Tissuevision) was used as previously described (Ragan et al., 2012; Y. Kim et al., 2017; Newmaster et al., 2020). Briefly, the brain was embedded in 4% oxidized agarose and cross-linked with 0.2% sodium borohydride solution. The brain was imaged as 12 × 16 × 280 tiles with 1 × 1 µm^2^ *x,y* pixel resolution in every 50-µm *z*-section. We used 910-nm wavelength for two-photon excitation to excite both green (e.g., eGFP) and red signals (e.g., tdTomato). Signals were separated with 560-nm dichroic mirror and two band path filters (607/70-25 for red and 520/35- 25 for green). Imaging tiles in each channel were stitched with custom-built software (Y. Kim et al., 2017; Newmaster et al., 2020).

For quantitative projection data analysis, we used our previously published pipeline (Jeong et al., 2016). Briefly, both signal and background channels were z-normalized. Then, the background channel images were subtracted from the signal channel images to increase signal-to-noise ratio. Then, projection signals were converted to a binary map by applying an optimized threshold (8× standard deviation) to detect signals while minimizing noise from background autofluorescence. Then, binarized signals in each pixel were counted in 20 × 20 (*x,y*) pixel unit (voxel) and the value was assigned the corresponding voxel across the brain, which is defined as “projection strength.” Thus, range of the projection strength in a given voxel is between 0 and 400. Projection strength of each area is calculated by summing up all projection strength within an anatomically defined area. Autofluorescence of brains was used to register each brain to the Allen CCF using Elastix (Klein et al., 2010), then, the projection signals were transformed to the reference brain. Then, we used maximum projection of registered long-range output datasets from each area to create a representative projection data for further quantitative analysis (Movie 2). “Area normalized projection” represents normalized occupancy of projection signals in the region of interest (ROI) by dividing the projection strength with a total number of voxels in each ROI. For example, if total voxel count for one ROI was 20,000 and our projection strength showed 2000 in the ROI, it will be (2000/20,000) × 100 = 10%. Regions with a projection strength >1% is designated as dense, between 1 and 0.5 as intermediate, between 0.5 and 0.1 as sparse, and <0.1 as very sparse (Fig. 2*E*).

Movie 1. Oxt neuronal expression in the whole brain. 10.1523/JNEUROSCI.0307-22.2022.video.1

For cell counting analysis, we used a machine-learning algorithm to detect fluorescently labeled cells (Y. Kim et al., 2017; Newmaster et al., 2020). The cell density in 2D (count/mm^2^) was calculated by dividing cell number with ROI area. 2D counting numbers were also converted into 3D counting using our previously calculated 3D conversion factor (1.4 for tdTomato; Y. Kim et al., 2017). To measure the volume of anatomic ROI, the reference Allen CCF was reverse registered onto individual brains using the Elastix. “Cell density (counts/mm^3^)” was calculated by dividing detected cell numbers in 3D with the anatomic ROI volume. The cell counting analysis was applied to Oxt-Cre;Ai14 and Oxtr-Venus cell distribution and inputs to the Oxt neurons. We used an average of individual datasets to create representative Oxt (Oxt-Cre;Ai14; Movie 1) and Oxtr (Oxtr-Venus; Movie 3) distribution and maximum projections to create monosynaptic input for Oxt neurons (rabies). For rabies input degree (Fig. 4*D*; Movie 4), Regions >100 cells are designated as dense, between 100 and 10 as intermediate, and <10 as sparse.

Movie 2. Brain-wide projection of Oxt neurons in the PVH and the SO. 10.1523/JNEUROSCI.0307-22.2022.video.2

Movie 3. Comparison between Oxtr expression and projection of hypothalamic oxytocin neurons. 10.1523/JNEUROSCI.0307-22.2022.video.3

Movie 4. Monosynaptic input to Oxt neurons in the PVH and the SO. 10.1523/JNEUROSCI.0307-22.2022.video.4

To compare relative abundance between Oxt output and Oxtr expression in Figure 3*C*, relative cell density or output data in each region was calculated by dividing each data by summed density or output data from all areas (excluding viral injection sites), respectively. Then, log10 (relative Oxt output/relative Oxtr) was used to examine the quantitative relationship between the two signals.

### 2D Hypothalamic and PVH flatmap

To generate the hypothalamic flatmap, we adapted the previously used method (Y. Kim et al., 2017) and applied it to the hypothalamic region. First, we created a binary image in the hypothalamic area based on the Oxt expression. Second, a zero line was placed to generate evenly spaced bins along the dorsal to the ventral direction of the PVH and laterally extended to include TU and MEA at different coronal plains. To capture signals on the flatmap, bins were registered into the reference brain and the cell number in each bin was quantified as described before in the STPT data analyses section. Lastly, the mean number of the Oxt neurons in eight Oxt-Cre;Ai14 brains were plotted in each flatmap using a custom-built MATLAB code. For the PVH flatmap, we followed the same procedure to generate a hypothalamic flatmap except for bin generation. Instead of delineating bins in a binary image, we assigned bin numbers in the PVH subregion of Franklin–Paxinos atlas (Paxinos and Franklin, 2008) in the dorsal to the ventral direction.

### Whole-brain clearing and immunostaining, LSFM, and cell counting

C57Bl/6 J mice [four males and three females at postnatal day (P)56] were transcardially perfused with 0.9% saline followed by 4% PFA in 0.1 m PB (pH 7.4). The decapitated heads were postfixed in 4% PFA overnight at 4°C and brains were dissected out the following day. All the following steps were performed on an orbital shaker unless otherwise specified. Dissected brains were delipidated in SBiP buffer (0.2 mm Na_2_HPO_4_, 0.08% SDS, 0.16% 2-methyl 2-butanol, 0.08% 2-propanol). Delipidation was performed with three to four washes (10 ml per wash) in SBiP for 24 h followed by one 10-ml wash with SBiP for the next 4 d. Samples were then moved to B1n buffer (0.1%v/v Triton X-100, 1%wt/v glycine, 0.001N NaOH, 0.008%wt/v sodium azide) for 1 d (10 ml) and then shifted to 37°C incubation for 3 h. Once delipidation was completed, the samples were washed in PTwH (Tween 20, 2 ml; 10 mg/ml Heparin, 1 ml; and sodium azide, 2 g, made to 1 l with 0.1 m PBS) three to five times at 37°C for 24 h. The samples were then incubated in antibody solution (5% DMSO and 3% donkey serum in PTwH- 4 ml per sample) containing primary antibodies for Oxt (rabbit polyclonal, ImmunoStar catalog #20068, RRID:AB_572258, 1:500) and Vasopressin (Guinea pig, polyclonal, Peninsula Laboratories catalog #T-5048, RRID:AB_2313978, 1:1000) for 10 d at 37°C. Next, PTwH washes were performed four to five times for 24 h at 37°C, followed by secondary antibody incubation. Secondary antibodies (1:500) were used as follows: Alexa Fluor 594 AffiniPure Fab Fragment Donkey Anti-Rabbit IgG (H+L; catalog #711-587-003, RRID: AB_2340623) and Alexa Fluor 647 AffiniPure F(ab')_2_ Fragment Donkey Anti-Guinea Pig IgG (H+L; catalog #706-606-148, RRID:AB_2340477) in antibody solution (4 ml per sample) for 10 d at 37°C. The samples were further washed three to four times in PTwH for 24 h at 37°C. Once immunolabeling was completed, the samples were moved to room temperature (RT) and further processed for tissue clearing. All the following steps were performed in a fume hood in glass containers and the containers were filled completely. Samples were dehydrated in the following series of methanol dilutions: 20%v/v, 1 h at RT; 40%v/v, 1 h at RT; 60%v/v, 1 h at RT; 80%v/v, 1 h at RT; 100%v/v, 1 h at RT; and 100%v/v at RT overnight. Next, the samples were incubated for 3 h in 66%v/v dichloromethane/33%v/v Methanol at RT followed by 100% dichloromethane (Sigma catalog #270997) incubations of 30 min and 2 h. Samples were then index matched in benzyl ether (Sigma catalog #108014) overnight without shaking. Once the samples are completely transparent (1–2 d), samples were moved to ethyl cinnamate (Sigma catalog #112372). Whole-brain samples were then imaged using a light sheet microscope (SmartSPIM, Life Canvas) at 2×2×5μm pixel resolution.

Oxt cell detection and 3D counting workflow are similar to the STPT based quantification by applying a 2-D fast fourier transform high pass filter, normalizing the data by dividing it by the filtered part, thresholding and 3D water-shedding to find the mask of each cell, and finally documenting each cell with its centroid location.

### Immunohistochemistry, microscopic image, and cell counting

For immunohistochemistry, fixed brains were either embedded in 3% agarose or frozen after sinking in 30% sucrose in 0.2 m PB. Embedded or frozen brains were then cut on a vibratome (Leica vt1000s) or a microtome (Leica SM2010 R) at 50-µm thickness. Sections were stored at −20°C in a cryoprotectant solution (30% sucrose and 30% glycerol in 0.1 m PB) until immunostaining. For Oxt staining, sections were washed three times in 1× PBS. After 1 h incubation in blocking solution (10% donkey serum and 0.1% Triton X-100), slices were incubated with Oxt primary antibody (rabbit polyclonal, ImmunoStar catalog #20068, RRID:AB_572258, 1:1000) in blocking solution for overnight at 4°C. Sections were then washed three times with 1× PBS and further incubated in secondary antibodies (Thermo Fisher Scientific catalog #A-21206, RRID:AB_2535792, 1:500) for 1 h at RT. After washing three times, slices were mounted onto slides with vectashield mounting media (Vector laboratories, H-1500-10). For microscopic imaging, a BZ-X700 fluorescence microscope (Keyence) and a confocal microscope (Zeiss 510) were used. A low magnification objective lens (4×) was used to image with a large enough view to define brain AP location from bregma and higher magnification objective lenses (10× ∼ 40×) were used to image sections depending on the cell density. Images were delineated manually based on the Franklin–Paxinos atlas and fluorescently tagged cells were manually quantified using the cell counter plug-in in FIJI (ImageJ, NIH).

### Software accessibility

All custom-built codes and flatmaps used in the current study will be freely available on request and can be used without any restriction.

### Data sharing plan

Data files for the anterograde projectome, rabies based monosynaptic input, and Oxtr expression data registered on the Allen CCF are available in https://kimlab.io/data_share/files/OT_mapping/.

High-resolution STPT images with web visualization is available in https://kimlab.io/brain-map/ot_wiring/.

## Results

### Quantitative density mapping of Oxt neurons reveals four clusters in the adult mouse brain

We first aim to determine quantitative brain-wide Oxt distribution in complex 3D structures. To examine the anatomic distribution of Oxt neurons across the whole brain, we used Oxt knock-in mice with Cre recombinase (Oxt-Cre) crossed with Ai14 reporter mice (Oxt-Cre;Ai14- heterozygotes; Choe et al., 2015). We imaged the entire mouse brain at cellular resolution using STPT and performed quantitative mapping using previously established computational methods (*n* = 8 brains; Fig. 1*A*,*B*; Movie 1; Table 1; Extended Data Table 1-1; Y. Kim et al., 2017). The PVH regions (PVH, descending division of PVH, anterior, intermediate, and subparaventricular zone) contain the highest density of Oxt neurons (∼39%, 742 out of total 1899 cells) followed by the tuberal nucleus (TU), SO, and other areas (Table 1). To further visualize the spatial expression pattern of Oxt neurons, we created a flatmap (Fig. 1*C*; abbreviation in Table 1). Evenly spaced bins provide a flattened 2D spatial unit to quantify and to display signals from the 3D brain. The flatmap was delineated with Allen CCF and Franklin–Paxinos atlas based anatomic labels (Fig. 1*C*,*D*; Paxinos and Franklin, 2008; Chon et al., 2019; Wang et al., 2020). The regional boundaries of the two labeling systems generally agreed with each other in the major Oxt-expressing regions (e.g., the PVH and the SO) despite noticeable discrepancies in the caudal hypothalamic area (e.g., the TU; Fig. 1*C*,*D*; Chon et al., 2019). The Oxt density heatmap on the hypothalamic flatmap clearly shows four clusters: (1) the PVH; (2) the SO; (3) accessory nuclei (AN); (4) the TU area (Fig. 1*E*; Knobloch and Grinevich, 2014). Notably, the largely overlooked TU area contains almost as high density of Oxt neurons as the PVH area (Fig. 1*E*).

10.1523/JNEUROSCI.0307-22.2022.f1-1 Extended Data Figure 1-1 Fluorescent images across 5 levels of the PVH, SO, and MEA. Genetically expressed Oxt neurons (Oxt-Cre;Ai14) are red and Oxt immunostaining cellar are labeled with green fluorescent marker are green. Scale bar: 50 µm. Download Figure 1-1, TIF file.

To distinguish neurons actively expressing Oxt in adults from developmentally labeled cells, we performed immunohistochemistry using an Oxt antibody in Oxt-Cre;Ai14 mice. We confirmed that almost all Oxt immuno positive neurons (97%, 1733 out of 1790 cells, *n* = 4 animals) were labeled by tdTomato from Oxt-Cre;Ai14 mice (Extended Data Fig. 1-1). In contrast, 76% of tdTomato labeled cells were Oxt immuno positive (1733 out of 2277 cells) in the PVH. Smaller portions of tdTomato cells in the SO (44%, 654 out of 1508 cells) and the MEA (8%, 31 out of 375 cells) retain active Oxt expression (Extended Data Fig. 1-1). This result suggested that Oxt neurons undergo Oxt expression changes during neurodevelopmental processes (Madrigal and Jurado, 2021).

To cross validate active expression of Oxt in the adult brain, we performed tissue clearing followed by 3D immunolabeling with Oxt and vasopressin antibodies in eight-week-old C57bl/6 mice (*n* = 7 brains; Fig. 1*F–I*; Renier et al., 2016). We developed 3D counting and image-registration methods to achieve similar unbiased brain-wide cell counting as done with STPT imaging (see Materials and Methods for more details). We observed similar Oxt staining distribution and overall slightly higher counting compared with our transgenic based mapping results (Table 1). For example, the estimated number of Oxt neurons in the PVH with the immunostaining was 1095 cells out of total 3149 cells (∼35%), which is higher than our transgenic based estimate mostly likely because of sensitive labeling based on antibody detection. Importantly, we confirmed the robust Oxt expression in the TU area that was not colocalized with vasopressin staining (Fig. 1*I*).

### Quantitative whole-brain projection mapping of Oxt neurons reveals broad long-range projections in nine functional circuits

Next, we aim to establish a comprehensive anterograde projection map from Oxt neurons in the four identified areas and examine whether Oxt projections target-specific functional circuits related to distinct behavior control.

Since Oxt can be released via axons, dendrites, and even neuronal processes (Jurek and Neumann, 2018), we injected a Cre-dependent adeno associated virus 2 (AAV2-CAG-Flex-EGFP) in the four areas of Oxt-Cre knock-in mice with slightly varying injection sites to cover target areas (*N* = 15 animals for the PVH, 10 for the SO, 2 for the AN, and 5 for the TU; Fig. 2*A*,*B*). We included male, virgin female, and lactating female mice in our study (see Table 2 and Materials and Methods for more detail), and observed no significant difference between sex or lactating state. Thus, we merged all data from the same anatomic areas. Long-range projection signals from individual injections were then registered onto the Allen CCF and maximum projection data in all samples from each anatomic area were used to represent efferent output for the four areas (Fig. 2*C*; Movie 2). We found abundant projections from Oxt neurons in the PVH to the midbrain and hindbrain areas while relatively sparse projection to the diencephalon and telencephalon areas (Fig. 2*D*)

**Table 2.** Nine functional circuits that are connected with Oxt neurons

Functional circuits	Full name	Abbreviation
Attention	Medial septal nucleus	MS
	Diagonal band nucleus	NDB
	Substantia innominata	SI
	Medial septal nucleus	MS
	Claustrum/dorsal endopiriform nucleus	CLA/EPd
	Locus ceruleus	LC
	Pedunculopontine nucleus	PPN
Threat, alert, defense	Lateral septal nucleus	LS
	Bed nuclei of the stria terminalis	BST
	Central amygdalar nucleus	CEA
	Basal medialy amygdala	BMA
	Parastrial nucleus	PS
	Dorsomedial nucleus of the hypothalamus	DMH
	Dorsal premammillary nucleus	PMd
	Periaqueductal gray, ventral lateral	PAG
	Midbrain reticular nucleus, retrorubral area	RR
	Midbrain reticular nucleus	MRN
Sleep/awake	Median preoptic nucleus	MEPO
	Ventrolateral preoptic nucleus	VLPO
	Suprachiasmatic nucleus	SCH
	Reticular thalamus	RT
	Supraoptic nucleus	SO
	Lateral preoptic area	LPO
	Subceruleus nucleus	SLC
	Retrochiasmatic area	RCH
	Paragigantocellular reticular nucleus, lateral part	PGRNl
	Pontine reticular nucleus	PRN
Pain	Subparafascicular area	SPA
	Subparafascicular nucleus, posterior	SPFp
	Periventricular hypothalamic nucleus, posterior part	PVp
	Peripeduncular nucleus	PP
	Parabrachial nucleus	PB
	Nucleus raphe magnus	RM
Sensory motor regulation	Auditory cortex	AUD
	Visual cortex	VIS
	Caudate putamen	CP
	Globus pallidus	GP
	Anterior olfactory nucleus	AON
	Piriform cortex	PIR
	Posterior intralaminar thalamic nucleus	PIL
	Zona incerta	ZI
	Subthalamic nucleus	STN
	Substantia niagra	SN
	Inferior colliculus, external nucleus	ICe
	Superior colliulus, motor	SCm
	Cuneiform nucleus	CUN
	Red nucleus	RN
	Pretectal region	PRT
	Pontine central gray	PCG
	Barrington's nucleus	B
	Gigantocellular reticular nucleus	GRN
	Superior olivary complex	SOC
	Parvicellular reticular nucleus	PARN
	Lateral reticular nucleus	LRN
	Facial motor nucleus	VII
	Spinal nucleus of the trigeminal, caudal part	SPVC
	Spinal nucleus of the trigeminal, interpolar part	SPVI
Body physiology/metabolism	Visceral cortex	VISC
	Gustatory cortex	GU
	Parasubthalamic nucleus	PSTN
	Posterior hypothalamic nucleus	PH
	Anterior hypothalamic nucleus	AHN
	Vascular organ of the lamina terminalis	OV
	Arcuate hypothalamic nucleus	ARH
	Lateral hypothalamic area	LHA
	Tuberal nucleus	TU
	Intermediate reticular nucleus	IRN
	Magnocellular reticular nucleus	MARN
	Medullary reticular nucleus	MDRN
	Nucleus ambiguus	AMB
	Parapyramidal nucleus	PPY
	Nucleus of the solitary tract	NTS
	Dorsal motor nucleus of the vagus nerve	DMX
	Area postrema	AP
Learning and memory	Field CA2	CA2
	Nucleus of reunions	RE
	Lateral mammillary nucleus	LM
	Rhomboid nucleus	RH
	Supramammillary nucleus	SUM
	Anterior group of dorsal thalamus	ATN
	Entorhinal area	ENT
	Tuberomammillary nucleus	TM
Reward, value assessment	Anterior cingulate area	ACA
	Infralimbic cortex	ILA
	Orbital cortex	ORB
	Agranular insular area	AI
	Nucleus accumbens	ACB
	Paraventricular nucleus of the thalamus	PVT
	Parataenial nucleus	PT
	Dorsal Raphe	DR
	Central linear nucleus raphe	CLI
	Interfascicular nucleus raphe	IF
	Ventral tegmental area	VTA
Reproductive	Medial preoptic nucleus	MPN
	Periventricular hypothalamic nucleus, preoptic part	PVpo
	Anteroventral periventricular nucleus	AVPV
	Medial preoptic area	MPO
	Anteroventral preoptic nucleus	AVP
	Ventral premammillary nucleus	PMv
	Ventromedial hypothalamic nucleus	VMH
	Posterodorsal preoptic nucleus	PD
	Medial amygdala	MEA

We then examined whether Oxt neurons in the four anatomic areas show any distinct projection pattern. Overall, the PVH neurons showed the broadest projection pattern followed by the SO and the AN, which project to a small subset of PVH-Oxt efferent areas (Fig. 2*D*,*E*; Movie 2). The TU-Oxt neurons did not show any long-range projections. We ask whether Oxt neurons project to distinct neural circuits related to specific function. Based on known functions of each anatomic region, we found that PVH-Oxt neurons project to three functional modules that control the internal state, somatic visceral, and cognitive response. Each module contains three circuits (Table 2 for abbreviations). The internal state module contains attention, threat/alert/defense, and sleep/awake circuits (Fig. 2*E*). The somatic visceral module includes pain, sensory motor, and body physiology/metabolism circuits (Fig. 2*E*). Lastly, the cognitive control module has learning/memory, reward/value assessment, and reproduction circuits (Fig. 2*E*). Each circuit is composed of multiple brain regions from the hindbrain, midbrain, thalamus, hypothalamus, cerebral nuclei, and cerebral cortex that process low-to-high order information. For instance, many basal ganglia circuit components including the caudate putamen (CP), globus pallidus (GP), subthalamic nucleus (STN), and substantia nigra (SN) receive Oxt projection to modulate motor function (see sensory motor regulation in Fig. 2*E*). PVH-Oxt neurons project to these areas at varying degrees. Dense projection occurs largely onto the hindbrain (e.g., the dorsal motor nucleus of the vagus nerve; DMX, the PB), the midbrain (the substantia nigra compacta; SNc), and the hypothalamus (the medial preoptic nucleus; MPN) to directly modulate motor output and sensory input (Fig. 2*E*). In contrast, the cerebrum (the cerebral nuclei and cerebral cortex) that works as high cognitive controller receives more sparse projection (Fig. 2*E*). The SO and AN project to a small subset of PVH-Oxt target areas (Fig. 2*E*). These data suggest that Oxt neurons in these two areas can further modulate a subset of the nine functional circuits, albeit less powerfully.

Together, our comprehensive projectome analysis uncovers anatomic substrates to explain pleiotropic effect of Oxt neurons regulating diverse behavioral outcomes.

### Oxtr expression showed quantitative mismatch with Oxt neuronal output

Next, we ask whether expression of a single subtype of the Oxtr is quantitively correlated with Oxt projection target areas to mediate circuit-specific Oxt function. Although most Oxt projecting areas are known to contain Oxtr expression (Grinevich et al., 2016), the quantitative relationship between Oxt projection and Oxtr expression across the whole brain is currently lacking.

To understand Oxt-Oxtr correlation, max Oxt projectome data from both the PVH and SO were compared with Oxtr expression in adult mice using a previously validated mouse line, Oxtr-Venus (Yoshida et al., 2009; Newmaster et al., 2020). A cohort of adult Oxtr-Venus mice brains were imaged using STPT and mapped Oxtr expression in the whole adult brain. These mapped Oxtr positive neurons (Fig. 3*A*, magenta) were registered onto the same reference brain along with Oxt-projections (Fig. 3*A*, green). Overall, the Oxtr showed high expression in the cortical area with minimal Oxt projection, while many midbrain and hindbrain regions have strong Oxt with little Oxtr expression (Fig. 3*A*,*B*; Movie 3). When we examined whether relative projection of Oxt neurons is correlated with relative Oxtr density across the entire brain, we found no significant correlation across the whole brain and major brain areas, except for the thalamus and the medulla (Fig. 3*C*). Overall, our results highlight lack of quantitative and spatial correlation between Oxt projections and Oxtr expression in the mouse brain.

Then, how do Oxtr rich areas (e.g., the isocortex) receive Oxt signaling without direct Oxt projection? A previous study suggested that many Oxtr-expressing neurons may receive Oxt signal non-synaptically via CSF (Zheng et al., 2014). Hence, we examined whether Oxt projection fibers make physical contact with ventricles. Indeed, we frequently found Oxt fibers with thick varicosities at the lateral, third, and fourth ventricle surface (Fig. 3*D*). This further suggests that Oxt signaling may transmit to the brain via the CSF route in addition to direct transmission in target areas.

### Oxt neurons mainly receive monosynaptic inputs from the thalamus, hypothalamus, and cerebral nuclei

Since Oxt neurons are known to integrate external stimuli and internal state, we ask whether Oxt neurons in the PVH and the SO receive monosynaptic input from sensory and integrative information processing brain areas.

To map brain-wide monosynaptic inputs in a cell type-specific manner, conditional retrograde pseudorabies viruses were injected into the PVH and the SO of the Oxt-Cre knock-in mice separately (Fig. 4*A*; Wickersham et al., 2007). We confirmed the specificity of labeling by performing co-immunolabeling avian tumor virus receptor A (TVA) positive neurons with Oxt and arginine vasopressin (AVP). None of the TVA infected neurons were AVP positive and were largely Oxt positive (Extended Data Fig. 4-1). We also confirmed no leakiness of TVA labeling by injecting TVA in the PVH of adult C57 mice which did not result in any infection (Extended Data Fig. 4-1). To confirm G protein dependency for monosynaptic tracing, TVA without G and rabies viruses were injected to the PVH and SO separately and the brains were imaged in STPT (*N* = 1 animal each for the PVH and SO). All the neurons observed were confined to the injection site and both samples were devoid of any long-range input cells. Lastly, we performed another rabies tracing experiment with optimized G and split TVA that are known for improved Cre specificity and tracing (E.J. Kim et al., 2016). We found near identical results with this alternative virus approach (Extended Data Fig. 4-2; *N* = 2 animals, each for the PVH and SO). Once we confirmed the validity of our input tracing methods, we used our mapping method to quantify input neurons throughout the whole brain (Y. Kim et al., 2017). To acquire overall inputs to each anatomic area, input signals from multiple independent injections targeting a specific brain region were registered onto the Allen CCF and the max projection of input signals from each anatomic area (*N* = 6 animals for the PVH and four for the SO) were overlaid onto the reference brain (pseudo-colored green for the PVH and magenta for the SO in Fig. 4*B*,*C*; Movie 4).

10.1523/JNEUROSCI.0307-22.2022.f4-1 Extended Data Figure 4-1 Oxt and Vasopressin immunolabelling in TVA-GFP injected Oxt-Cre mice. A, TVA-GFP neurons are co-localized with Oxt (arrows), whereas no vasopressin positive neurons co-localized with TVA-GFP. TVA-GFP labeled in green, Oxt in red and vasopressin in blue. B, Specificity of TVA-GFP to infect only Cre positive neurons. TVA-GFP co-injected with CAG-tdTomato in the PVH of C57 mice. TVA-GFP labeled in green and CAG-tdTomato labeled in red. Scale bar: 200 µm. Download Figure 4-1, TIF file.

10.1523/JNEUROSCI.0307-22.2022.f4-2 Extended Data Figure 4-2 Pseudorabies tracing experiments using split TVA and optimized G injections in the PVH and SO of Oxt-Cre mice. A, oG-nls-mCherry marked in red and rabies GFP labeled as green, with overlapping neurons visualized as yellow. B, High-magnification images showing cell bodies of neurons from which PVH and SO Oxt neurons receive monosynaptic inputs. Scale bar: 200 µm. Download Figure 4-2, TIF file.

Overall, Oxt neurons from the PVH mainly receive inputs from the thalamus, hypothalamus, and cerebral nuclei (Fig. 4*C*). All brain regions providing inputs to the Oxt neurons also receive output from the Oxt neurons except the triangular nucleus of septum (TRS), creating reciprocal connections with afferent areas (Figs. 2*E*, 4*D*). Noticeably, Oxt neurons received little input from hindbrain despite strong output to the same area, suggesting that Oxt neurons provide largely unilateral output to the hindbrain (Figs. 2*E*, 4*D*). Moreover, the cerebral cortex provides little to no input to the Oxt neurons, further supporting very weak direct interaction between cerebral cortical areas and Oxt neurons (Fig. 4*D*; Movie 4).

SO-Oxt neurons receive overall similar input compared with the PVH-Oxt neurons (Fig. 4*D*). The broad afferent pattern is in sharp contrast to the very sparse efferent projection of SO-Oxt neurons to the brain (Figs. 2*E*, 4*D*). When monosynaptic input from the PVH-Oxt and SO-Oxt neurons are compared, SO-Oxt neurons show input from relatively more lateral parts of the brain (Fig. 4*C*, arrows).

Collectively, we conclude that Oxt neurons in the PVH and the SO receive similar input from a subset of brain areas that receive majority of input from hypothalamic areas followed by cerebral nuclei and thalamic areas (Fig. 4*D*).

### Input-output wiring diagrams of PVH-Oxt and SO-Oxt neurons provide overall neural circuit control patterns

**Figure 5.**
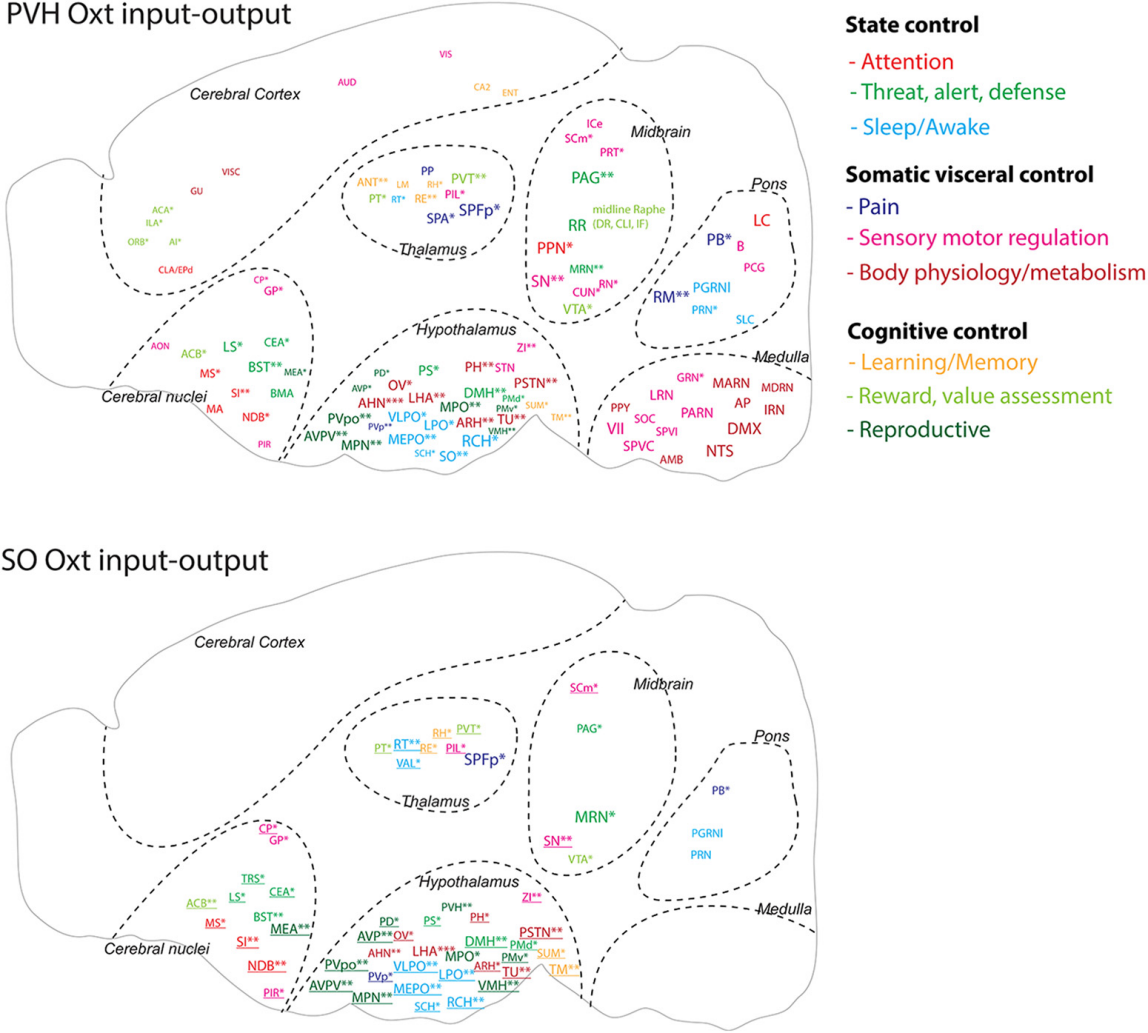
Oxt neuron input-output wiring diagram. Schematic summary of monosynaptic input and axonal output connectivity of Oxt neurons. Color of each ROI is related to nine functional circuits. ROI size is correlated with Oxt output degree as done in Figure 2. Areas providing the monosynaptic input are highlighted with asterisk (*). Number of * indicated input strength: * < 10 cells, 10 ≤ ** < 100, 100 ≤ ***. Monosynaptic input areas without receiving Oxt output in the SO-Oxt map were indicated with underlined fonts. All abbreviations for brain regions can be found in Table 2.

Based on our long-range output and monosynaptic input data, we constructed input-output circuit diagrams of Oxt neurons in the PVH and the SO while annotating each brain area based on their functional categories (Fig. 5; Table 2 for abbreviations). PVH-Oxt neurons project broadly to all nine identified functional circuits throughout the brain, indicating that Oxt neurons can modulate information processing at different level of circuits with overall stronger influence in the midbrain and hindbrain circuits (Fig. 5). In contrast, mid-level circuits including the diencephalon (the thalamus, hypothalamus), the midbrain, and the cerebral nuclei, provide major input to inform action of Oxt neurons, providing anatomic substrate to perform an integrative role (Fig. 5). SO-Oxt neurons receive similar mid-level circuit input compared with the PVH-Oxt neurons while showing limited central projection to the midbrain and pons (Fig. 5). This suggests that SO-Oxt neurons mainly serve as peripheral hormonal output.

## Discussion

The wiring diagram of the brain is a structural foundation to decipher neural circuits underlying brain function. Here, we present a comprehensive anatomic connectivity map of the hypothalamic Oxt neurons and their relationship with postsynaptic Oxtr expression in the whole mouse brain. A quantitative mismatch exists between Oxt projection and Oxtr distribution pointing toward abundant non-synaptic Oxt signaling within the brain. We also identify nine functional circuits with reciprocal or unidirectional connection with Oxt neurons that serve as anatomic entities to exert varied behavioral control.

Oxt neurons are mostly located in hypothalamic nuclei with a complex 3D shape (Biag et al., 2012; Madrigal and Jurado, 2021). To examine Oxt expression intuitively and quantitatively, we devised a 2D flatmap for Oxt containing hypothalamic regions from an Allen CCF-based reference brain while incorporating anatomic labels from the Allen Institute and Franklin–Paxinos (Paxinos and Franklin, 2008; Wang et al., 2020). This approach allows for the interpretation of Oxt anatomic location from two independently created and commonly used atlases (Chon et al., 2019) and provides an alternative coordinate system to understand anatomic connectivity. Oxt neuronal cell counts remain similar across male, virgin female and lactating female mice, which is consistent with an earlier study in rats (Knobloch et al., 2012). This suggests that secreted Oxt level per cell may change based on physiological conditions (e.g., lactation) without changing Oxt cell numbers in normal mice, although reduction of Oxt neurons has been reported in animal models of neurodevelopmental disorders (Peñagarikano et al., 2015; Dai et al., 2018). In addition to well-described Oxt neurons in the PVH, SO, and AN, we described another major population in the TU area in the hypothalamus (Jirikowski, 2019). Our 3D immunolabeling independently validated the existence of this extra population. Our anterograde tracing showed that these neurons have almost no central projection, suggesting their contribution to brain information processing is limited. Future studies including ablation studies will help to elucidate the functional significance of this overlooked Oxt population.

Oxt signaling is known to modulate many distinct brain functions such as anxiolytic effect, social memory, and attention (Lee et al., 2009; Marlin et al., 2015; Grinevich and Stoop, 2018; Schiavo et al., 2020). By extensively mapping Oxt efferent processes and clustering brain regions based on known functions, we identified nine functional circuits where Oxt processes interact to modulate distinct behavioral circuits. Each circuit consists of a set of brain regions processing different behavioral aspects. Thus, our circuit map can help to understand neural entities of Oxt that modulate different behavioral aspects. Overall, Oxt circuits provide broad projections to modulate external and internal information throughout the entire brain circuit. For example, we found that Oxt neurons project to sensory-motor and pain circuits from the hindbrain and midbrain to cerebral cortex and cerebral nuclei. A recent single cell reconstruction study demonstrated that even a single magnocellular Oxt neuron can make multiple collateral projections to extrahypothalamic areas to coordinate neuromodulation across functionally related brain circuits (Zhang et al., 2021). These provide anatomic evidence that Oxt neurons can finely modulate sensory motor processing throughout different circuit levels. Notably, Oxt neurons project to other neuromodulatory areas such as the locus coeruleus for norepinephrine (alert), the substantia nigra (movement) and the ventral tegmental areas for dopamine (reward), and raphe nuclei for serotonin (emotion), thus serving as a master neuromodulator of neuromodulations (Yoshida et al., 2009; Dölen et al., 2013; Hung et al., 2017; Froemke and Young, 2021). The most well-established role of Oxt signaling is to promote social behavior (Kemp and Guastella, 2010; Shamay-Tsoory and Abu-Akel, 2016). Social behavior is a complex behavior, requiring coordinated interplay between the sensory system and integrative circuits to generate socially appropriate motor outputs. Extensive connections of Oxt neurons to somatic visceral, cognitive, and state control modules can help to fine-tune activity of different circuit components to generate enhanced response to socially salient stimuli.

Oxt gets released through axonal and dendritic projections based on the inputs that Oxt neurons receive. The presence of large dense core vesicles containing Oxt at the nonactive zones of presynapses (Theodosis, 1985; Griffin et al., 2010), absence of evidence for Oxtr in the postsynaptic membranes, and extremely delayed electrophysiological Oxt response (milliseconds to seconds; Knobloch et al., 2012; Knobloch and Grinevich, 2014) collectively support non-synaptic axo-dendritic release of Oxt (Oti et al., 2021). Hence, our projection maps with entire process labeling provide possible release sites of Oxt throughout the whole brain. We also compared Oxt total projections (combined data from the PVH-Oxt and SO-Oxt neurons) to Oxtr expression in the central brain. Although earlier studies mentioned Oxt-Oxtr discrepancy, recent studies showed that most Oxtr-expressing areas contain at least sparse Oxt projection (Knobloch et al., 2012; Grinevich et al., 2016; Mitre et al., 2016; Zhang et al., 2021). Despite a few areas with high levels of both Oxtr and Oxt projection (e.g., the paraventricular thalamus), our analysis revealed no significant quantitative correlation between Oxt and Oxtr across entire brain regions. For example, the cerebral cortical area contains abundant Oxtr with little to no Oxt axons. However, Oxt can still mediate sensory stimuli in the cortex to modify mouse behavior (Marlin et al., 2015; Schiavo et al., 2020). Previous studies suggest that Oxtr neurons in the isocortex may receive Oxt signals indirectly from ventricular pathways via cerebral spinal fluid with delayed and long-lasting effects (Mens et al., 1983; Zheng et al., 2014). Indeed, we found that long-range processes from Oxt neurons contact the ventricle surface, suggesting potential release of Oxt into the CSF via long-range processes. Another noteworthy Oxt-Oxtr discrepancy is brain regions with abundant Oxt projection without Oxtr expression such as sensory related hindbrain areas. Although Oxtr is a main Oxtr, Oxt can bind to another receptor to exert its effect. For example, Oxt can elicit TRPV1 activity in the spinal cord to modulate nociception (Nersesyan et al., 2017). Oxt modulation in the central nervous system through these noncanonical pathways are under explored and requires further study.

Our Oxt monosynaptic input maps showed that the majority of inputs are from the cerebral nuclei, thalamus, hypothalamus, and midbrain with little input from the hindbrain. Particularly, almost all afferent brain regions to PVH-Oxt neurons also receive efferent projections, suggesting strong reciprocal control of target regions by PVH-Oxt neurons except the hindbrain for unidirectional output. Abundant bidirectional connections with nine functional circuits suggest that PVH-Oxt neurons can be an allostatic tool to interactively orchestrate and facilitate social and nonsocial information processing based on external stimuli and internal state (Quintana and Guastella, 2020). In contrast, despite having similar afferent areas to SO-Oxt neurons, their limited central projection suggests that SO-Oxt neurons serve largely as unidirectional hormonal output to the periphery rather than reciprocal circuit modulator. Although not included in the current study, examining monosynaptic input in the TU-Oxt neurons in the future can provide potential functional roles of the overlooked Oxt population.

In summary, our study provides an anatomic foundation to understand diverse functions based on Oxt neurons in the brain. We deposit all high-resolution imaging data in publicly accessible databases and our website to facilitate data mining. We envision that this Oxt wiring diagram with quantitative expression data will guide future studies to understand circuit-based mechanisms of Oxt function and its changes in socially relevant behaviors as well as brain disorders such as autism.
