# Supplementary material for: Whole-Brain Wiring Diagram of Oxytocin System in Adult Mice
Source: J Neurosci. 2022 Jun 22;42(25):5021–33. doi: 10.1523/JNEUROSCI.0307-22.2022 (PMC9233446; doi:10.1523/JNEUROSCI.0307-22.2022)
Supplement: Extended Data Table 1-1 — Comparison of Oxt cell counts across males, virgin females, and lactating females Download Table 1-1, DOCX file. [file ns-JN-RM-0307-22-s08.docx]

Table 1-1: Comparison of Oxt cell counts across males, virgin females and lactating females

| **Full Names** | **Abbreviations** | **Total (N=8)** | **Males (N=3)** | **Virgin Females (N=3)** | **Lactating Females (N=2)** |
| --- | --- | --- | --- | --- | --- |
| Paraventricular hypothalamic nucleus | PVH | 511.5 ± 147.1 | 562.1 ± 38.6 | 553.9 ± 189 | 435.4 ± 159.3 |
| Periventricular hypothalamic nucleus, anterior part | Pva | 47.6 ± 22.7 | 51.8 ± 2 | 56 ± 32.8 | 36.4 ± 20.2 |
| Periventricular hypothalamic nucleus, intermediate part | Pvi | 9.6 ± 11.4 | 9.8 ± 4 | 17.3 ± 16.2 | 1.9 ± 2.1 |
| Subparaventricular zone | SBPV | 17.9 ± 20.8 | 14.7 ± 1 | 30.3 ± 33.2 | 7.5 ± 2.9 |
| Periventricular hypothalamic nucleus, preoptic part | PVpo | 51.1 ± 18.3 | 71.4 ± 13.9 | 53.2 ± 6.4 | 35.5 ± 15.7 |
| Paraventricular hypothalamic nucleus, descending division | PVHd | 153.8 ± 49.7 | 154 ± 23.8 | 170.3 ± 61.4 | 137.2 ± 61.4 |
| Supraoptic nucleus | SO | 202.3 ± 65.5 | 214.9 ± 70.3 | 167.1 ± 32.4 | 229.1 ± 91.6 |
| Tuberal nucleus | TU | 472.9 ± 65.2 | 525.7 ± 18.8 | 444.7 ± 37.3 | 465.7 ± 96.2 |
| Medial amygdalar nucleus | MEA | 108.7 ± 49.6 | 160.3 ± 10.9 | 70 ± 55.8 | 112.9 ± 22.2 |
| Bed nuclei of the stria terminalis | BST | 27.8 ± 11 | 35.7 ± 8.9 | 32.2 ± 10.1 | 18.2 ± 7.4 |
| Substantia innominata | SI | 6 ± 3.1 | 4.2 ± 2 | 7.9 ± 3.5 | 5.1 ± 2.9 |
| Arcuate hypothalamic nucleus | ARH | 147.5 ± 86.5 | 219.1 ± 20.8 | 90.5 ± 94.1 | 156.8 ± 83.4 |
| Suprachaismatic nucleus | SCH | 1.2 ± 1.2 | 2.1 ± 1 | 1.4 ± 1.4 | 0.5 ± 0.8 |
| Ventrolateral preoptic nucleus | VLPO | 21.9 ± 23.3 | 16.8 ± 15.8 | 11.2 ± 4.8 | 35.9 ± 35.5 |
| Anterior hypothalamic nucleus | AHN | 21.9 ± 11.8 | 29.4 ± 13.9 | 27.1 ± 11.4 | 11.7 ± 2.9 |
| Medial preoptic nucleus | MPN | 18 ± 5.8 | 22.4 ± 0 | 17.3 ± 3.2 | 15.9 ± 9 |
| Lateral hypothalamic area | LHA | 45.9 ± 14.4 | 56 ± 2 | 52.3 ± 13 | 32.7 ± 11.3 |
| Lateral preoptic area | LPO | 2.1 ± 2 | 3.5 ± 1 | 0.9 ± 0.8 | 2.3 ± 2.9 |
| Retrochiasmatic area | RCH | 8.9 ± 6.6 | 16.8 ± 0 | 6.1 ± 5.3 | 6.5 ± 6.5 |
| Zona incerta | ZI | 19.8 ± 11.1 | 19.6 ± 9.9 | 21.9 ± 10.3 | 17.7 ± 16.2 |

Oxt-Cre:Ai14C cell counts from males, females and lactating females. Counting data are mean ± standard deviation
