# Supplementary figures and images for: Whole-Brain Wiring Diagram of Oxytocin System in Adult Mice
Source: J Neurosci. 2022 Jun 22;42(25):5021–33. doi: 10.1523/JNEUROSCI.0307-22.2022 (PMC9233446; doi:10.1523/JNEUROSCI.0307-22.2022)

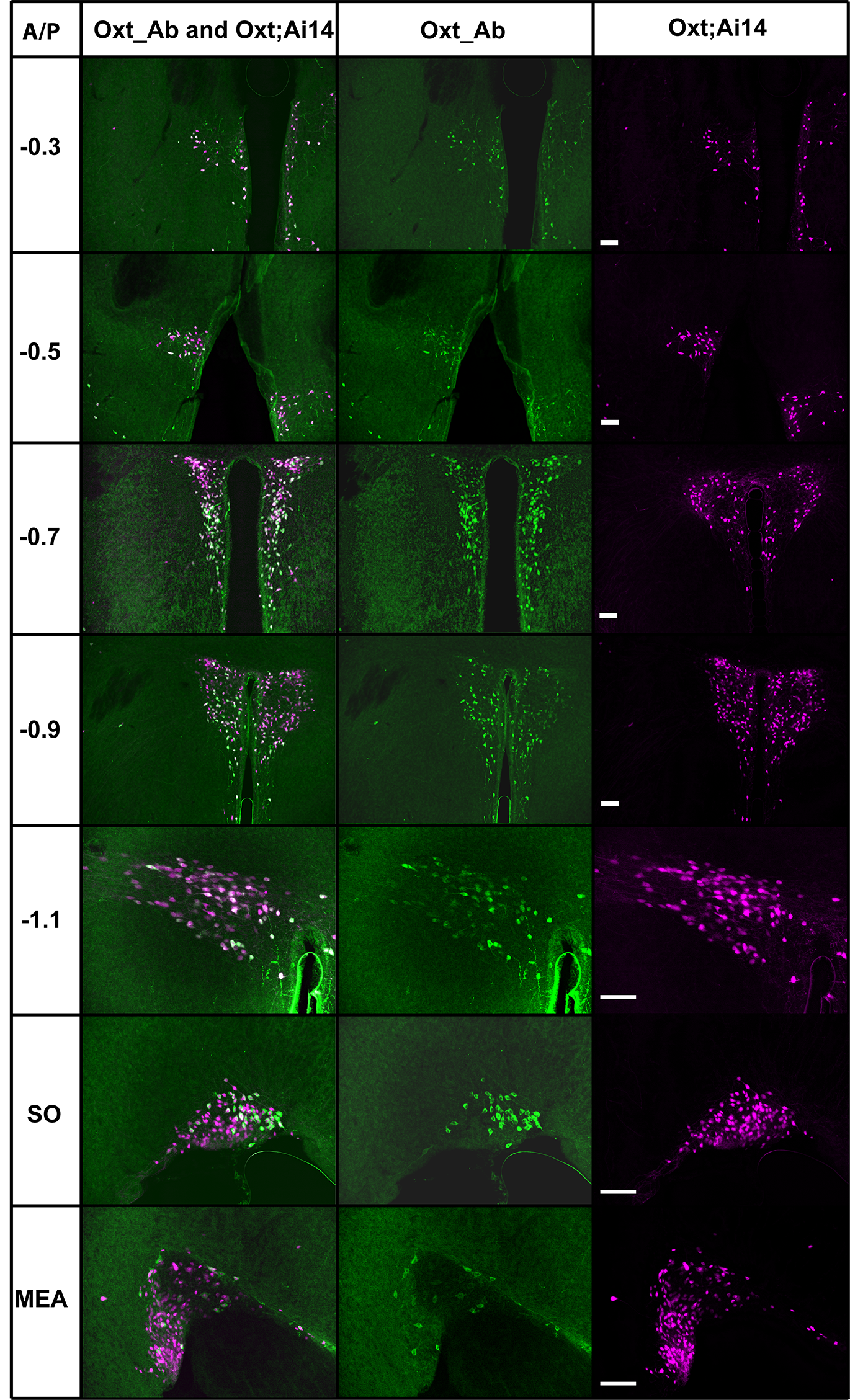

Supplement: Extended Data Figure 1-1 — Fluorescent images across 5 levels of the PVH, SO, and MEA. Genetically expressed Oxt neurons (Oxt-Cre;Ai14) are red and Oxt immunostaining cellar are labeled with green fluorescent marker are green. Scale bar: 50 µm. Download Figure 1-1, TIF file. [file ns-JN-RM-0307-22-s05.tif]

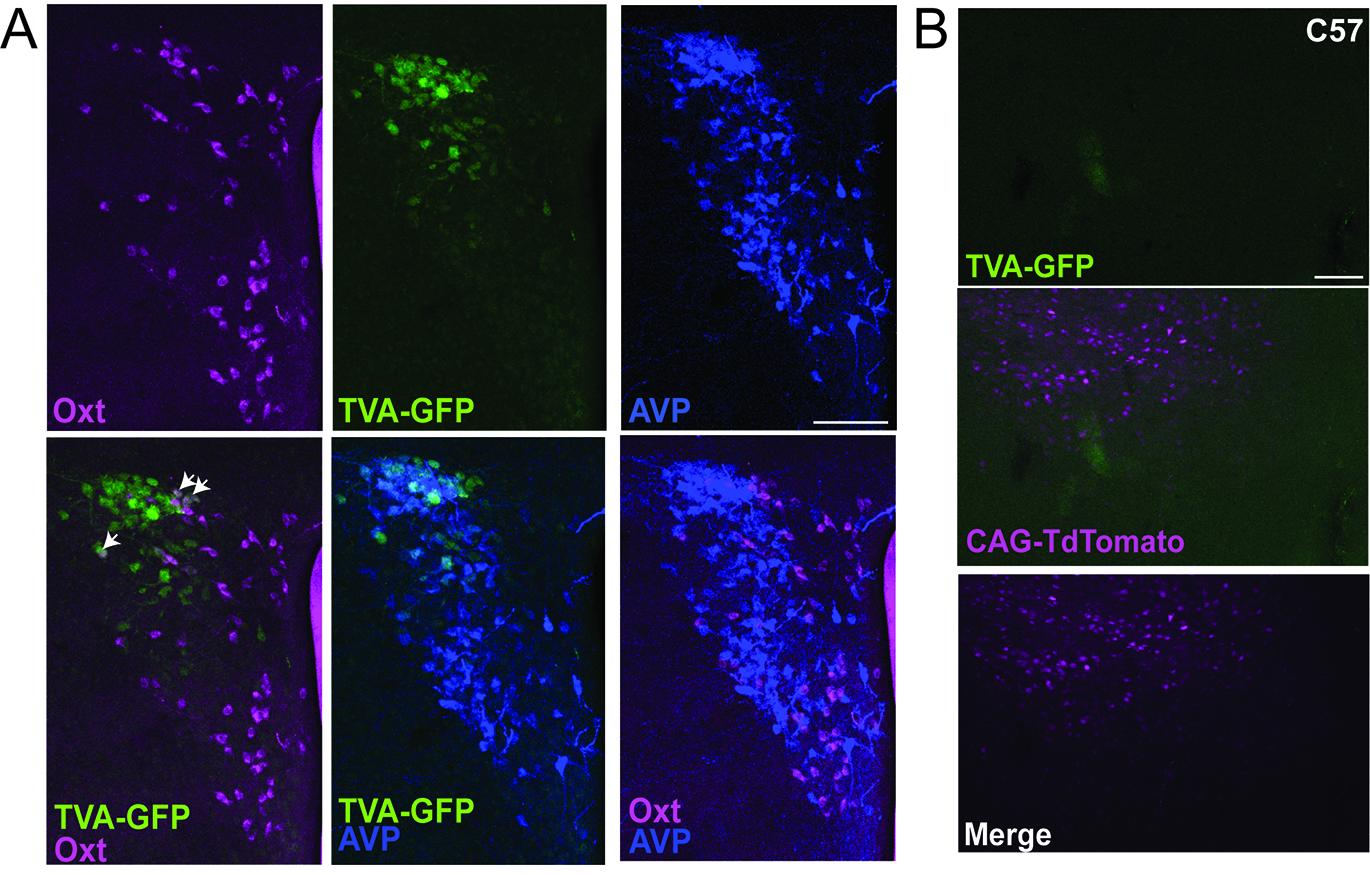

Supplement: Extended Data Figure 4-1 — Oxt and Vasopressin immunolabelling in TVA-GFP injected Oxt-Cre mice. A, TVA-GFP neurons are co-localized with Oxt (arrows), whereas no vasopressin positive neurons co-localized with TVA-GFP. TVA-GFP labeled in green, Oxt in red and vasopressin in blue. B, Specificity of TVA-GFP to infect only Cre positive neurons. TVA-GFP co-injected with CAG-tdTomato in the PVH of C57 mice. TVA-GFP labeled in green and CAG-tdTomato labeled in red. Scale bar: 200 µm. Download Figure 4-1, TIF file. [file ns-JN-RM-0307-22-s06.tif]

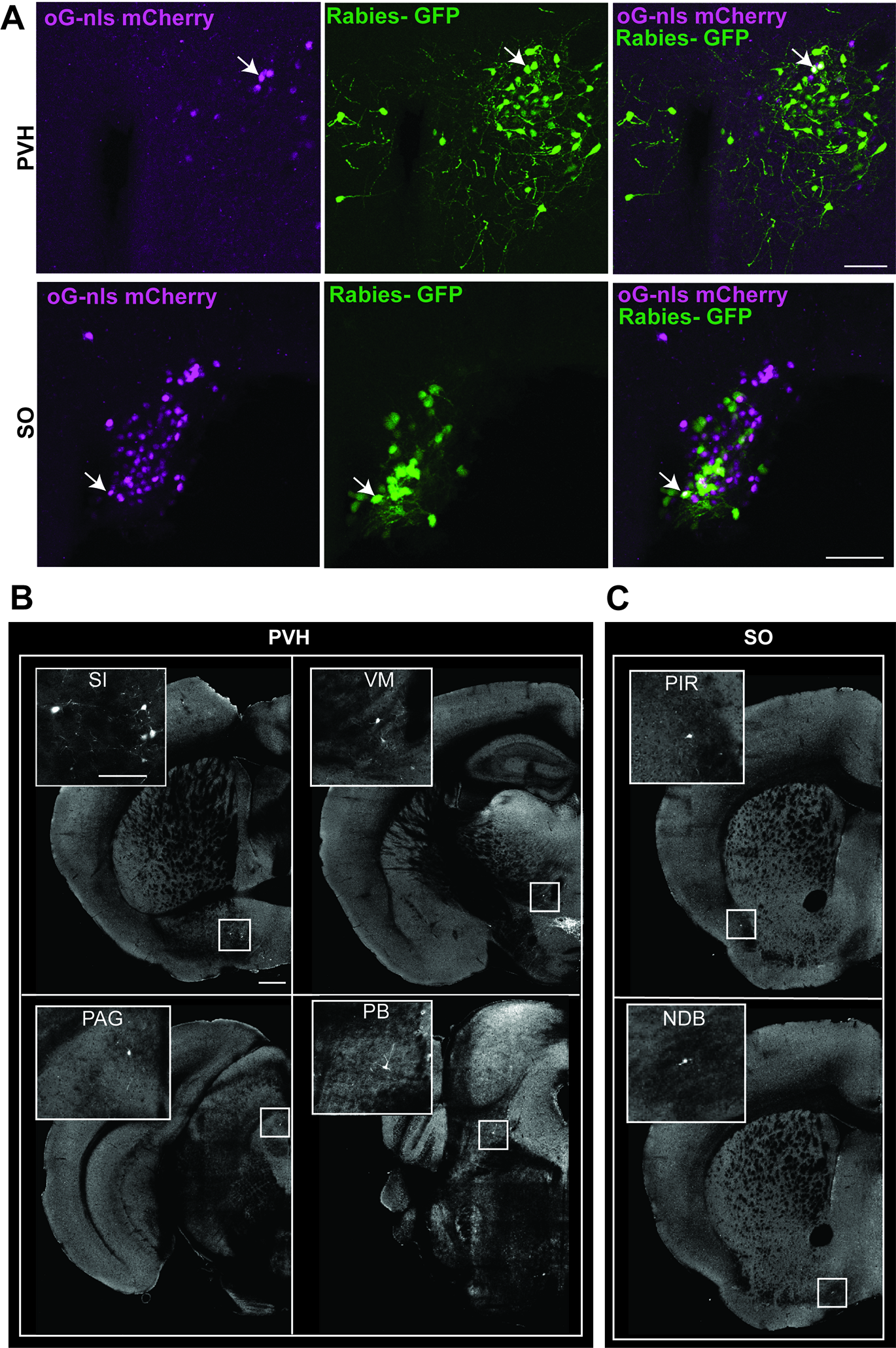

Supplement: Extended Data Figure 4-2 — Pseudorabies tracing experiments using split TVA and optimized G injections in the PVH and SO of Oxt-Cre mice. A, oG-nls-mCherry marked in red and rabies GFP labeled as green, with overlapping neurons visualized as yellow. B, High-magnification images showing cell bodies of neurons from which PVH and SO Oxt neurons receive monosynaptic inputs. Scale bar: 200 µm. Download Figure 4-2, TIF file. [file ns-JN-RM-0307-22-s07.tif]
